# COVID-19: critical case of a patient with an atypical manifestation of the disease

**DOI:** 10.1186/s13000-023-01289-w

**Published:** 2023-01-12

**Authors:** Eira Valeria Barrón-Palma, Siddhartha Ríos-Zaragoza, Ana Laura Sanchez-Sandoval

**Affiliations:** 1 Servicio de Medicina Genómica, Hospital General de México, Calle Dr. Balmis # 148, Colonia Doctores, Delegación Cuauhtémoc, C.P. 06726 México City, México; 2 Asistentes Dirección General, Hospital General de México, México City, México

**Keywords:** SARS-CoV-2, COVID-19, Idiopathic chronic ulcerative colitis, Multiple organ dysfunction syndrome, Viral mutation

## Abstract

**Background:**

COVID-19 was initially described as a severe acute respiratory disease that could drive to pneumonia, compromising the life of the patients in the worst scenario. However, even though in most of the cases the respiratory symptoms are still the most common manifestations of the disease, nowadays it is considered as a complex multisystem illness, affecting a variety of organs and tissues. Asymptomatic and atypic cases have also been described, where symptoms are not related to those first described, as is the case of this report.

**Case presentation:**

On November 23, 2020, a 53-year-old woman goes to the emergency room due to gastrointestinal symptoms. The admission diagnosis was inflammatory bowel disease and a mild event of idiopathic chronic ulcerative colitis, and the initial treatment was focused on the metabolic acidosis, and the reestablishment the hydroelectrolytic and hemodynamic balance. Then, she was transferred to the Gastroenterology Unit where she was treated for one week. During her hospitalization, she showed a refractory shock caused by progressive organ deterioration (renal and neurological), requiring a double-vasopressor support, oxygenation, and ventilation. Considering the laboratory tests results and computed tomography scans, a COVID-19 test was carried out, obtaining a positive result with a high viral load. The S gene of the virus was amplified and sequenced, finding an uncommon mutation rarely reported worldwide. After considerable systemic deterioration, the patient presented cardiorespiratory arrest, with no response and died on December 1, after 8 days of hospitalization.

**Conclusions:**

In this report we describe the pathogenesis, clinical manifestations, and outcome of a patient with atypical COVID-19 symptoms (mainly gastrointestinal), rapidly evolving and with lethal consequences. Therefore, it is important to emphasize the need to strengthen patient surveillance in health centers, including those who do not present typical symptoms of COVID-19**. **In addition, it will be important to track the identified mutation (H1058Y) in the S viral gene and assess whether it could be associated with a different clinical manifestation of the disease or if it was just an isolated event.

## Background

In December 2019, a novel human infectious disease emerged in Wuhan, China, beginning with an atypical pneumonia with an unknown cause [1]. As the disease rapidly spread, it was possible to isolate and identify the causal agent of the coronavirus disease 19 (COVID-19): a new coronavirus named Severe Acute Respiratory Syndrome Coronavirus 2 (SARS-CoV-2) [2]. Initially, it was considered solely as a respiratory infection with a variety of presentations, from mild respiratory tract infection to severe pneumonia and acute respiratory distress syndrome, but over the time, and due to the exponentially increased number of infections, it became evident that other organs and tissues are also affected by this virus.

The S protein of SARS-CoV-2 is responsible for the recognition of the host cell, as it binds to the angiotensin-converting protein 2 (ACE-2) of the target cell, mediating the entry of the virus [3]. ACE-2 receptors are expressed in a large number of tissues, including the lung epithelium, nasal mucosa, kidneys, heart, and small intestine, which facilitates extrapulmonary infection by SARS-CoV-2, causing a wide variety of complications in different organs [4].

Mutations on the S viral gene, specifically on the receptor binding domain (responsible of the physically interaction with ACE-2) can potentially determine a differential affinity for this receptor or other tissues, and consequently, possible different clinical manifestations [5].

## Case presentation

A 53-year-old woman began with gastrointestinal symptoms on November 15, 2020: Bristol type 7 stool evacuations, without mucus or blood, in an amount of 2 per day (approximately 100 ml per occasion), with no food remains, without urgency, and with fecal incontinence. She declared to be a smoker (suspended 6 years ago), not an alcoholic, and having had a transfusion on November 2019 (due to severe anemia). The patient also reported having been diagnosed with Idiopathic Chronic Ulcerative Colitis (ICUC) in November 2019, after presenting episodes of intermittent diarrhea of ​​2 years of evolution (10 depositions/day). She was medicated with mezalazine, cholecalciferol, omega 3 and omeprazole.

On November 23, 2020, she goes to the emergency room due to an increase in the number and volume of depositions, probably associated with the suspension of the treatment (referred by a family member). Initial exploration showed a frank wasting syndrome, apparent age of the patient equal to true chronological age. Oriented, Glasgow Coma Scale: 15. Oral mucosa with a regular state of hydration. Chest with conserved movements, with no pleuropulmonary syndrome. Abdomen without visceromegaly. Rectal examination was performed with difficulty due to anal pain, hypertonic sphincter, and traces of fresh blood in evacuations were found, and no lumps were palpated.

The admission diagnosis was inflammatory bowel disease and a mild event of ICUC (Table 1. Admission diagnosis). The patient met the criteria for hemodynamic instability, hence, hydroelectrolytic replacement began, without requiring support with amines. Once stabilized, she was transferred to the Gastroenterology Unit.

**Table 1:** Admission diagnosis

1. Inflammatory bowel disease. Idiopathic Chronic Ulcerative Colitis Mild Outbreak Montreal E3^a^, Truelove 13 points^b^
2. Hypovolemic shock Referred secondary to: - Acute diarrheal syndrome - Low intake
3. Water and electrolyte imbalance without electrocardiographic translation. - Hypokalemia - Hypocalcemia
4. Acute Kidney Injury AKIN^c^ 2
5. High Anion GAP Metabolic Acidosis
6. Malnutrition - Moderate CONUT^d^ - BMI^e^ 16.6

^a^Montreal classification of extent of ulcerative colitis

^b^Truelove-Witts index of ulcerative colitis activity

^c^AKIN: Acute Kidney Injury Network

^d^CONUT: Controlling Nutritional status

^e^BMI: Body Mass Index

Considering the clinical background and the initial evaluation at the emergency room, the treatment strategy was focused on the gastrointestinal symptoms and the reestablishment the hydroelectrolytic and hemodynamic balance. Daily laboratory tests were performed, as well as a variety of clinical examinations. A marked metabolic acidosis (pH = 6.8) by the time of the admission stands out, which gradually decreased through day 7 of hospitalization (pH = 7.2). Regarding serum electrolytes, she showed hyperphosphatemia (4.6–5.1 mg/dl), hyperchloremia (109–129 mg/dl), and hypocalcemia (4.5–6.9 mg/dl) as well as intermittently hypokalemia, during the whole time that she remained hospitalized; and hypernatremia in the last three days of hospitalization.

**Fig. 1:**
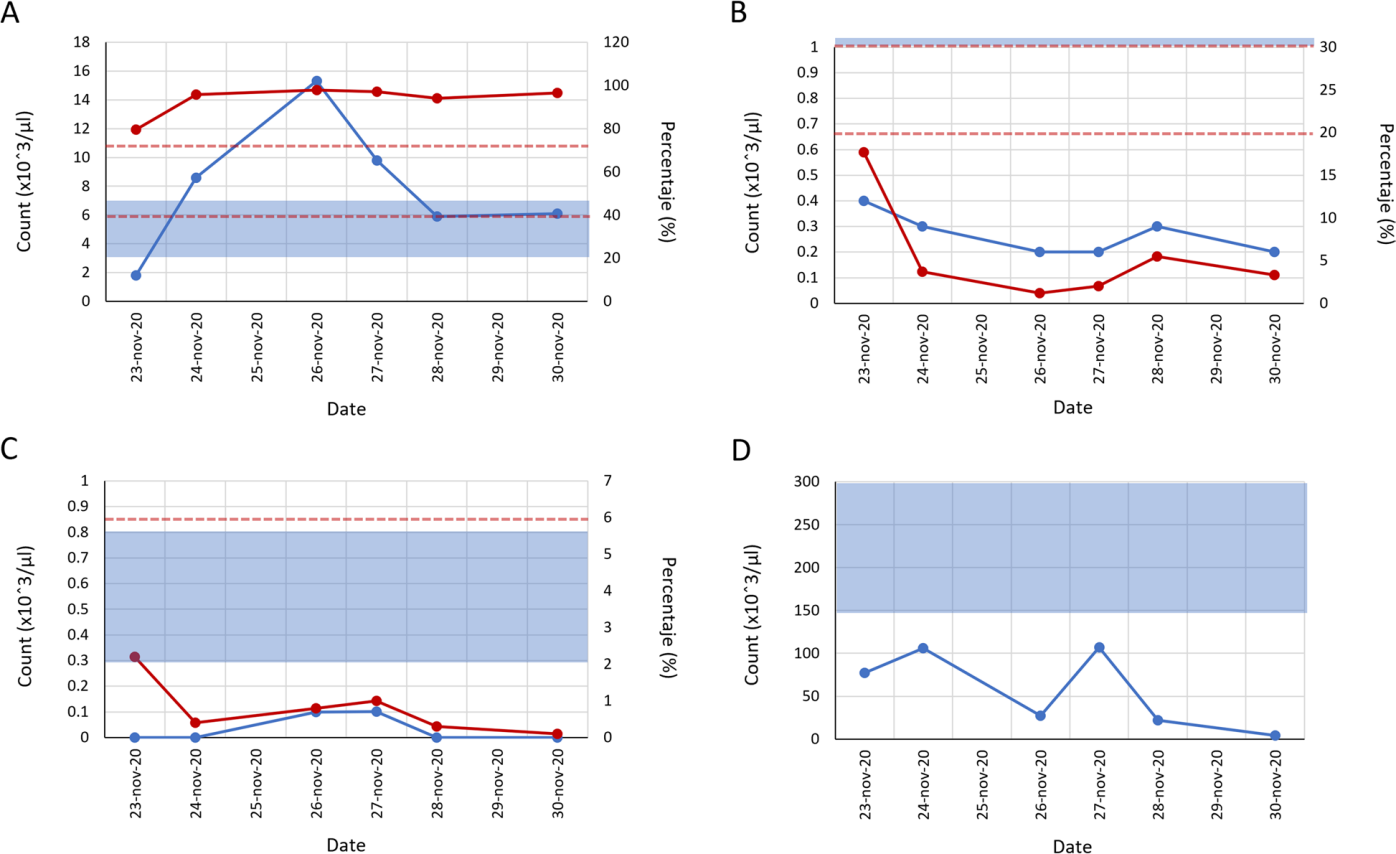
Blood count tests. The graphs of different measurements of blood cells during the time of hospitalization of the patient are shown. **A** neutrophils, **B** lymphocytes, **C** monocytes, and **D** platelets. Cell number (blue line) and percentage (red line) values ​​are shown; the solid blue bar and the interval between the red dotted lines represent the reference values ​​for cell count and percentage, respectively

Blood count tests showed altered profiles during the whole time of hospitalization (Fig. 1), highlighting an elevated percentage of neutrophils, although in the cell count, a clear increase is observed only between days 2 and 5 of hospitalization (Fig. 1 A). ​​In total lymphocytes and monocytes, both cell count and percentage levels, were always lower than the reference values (Fig. 1B C), in addition, low number of total platelets was detected throughout the hospitalization time (Fig. 1D).

**Fig. 2:**
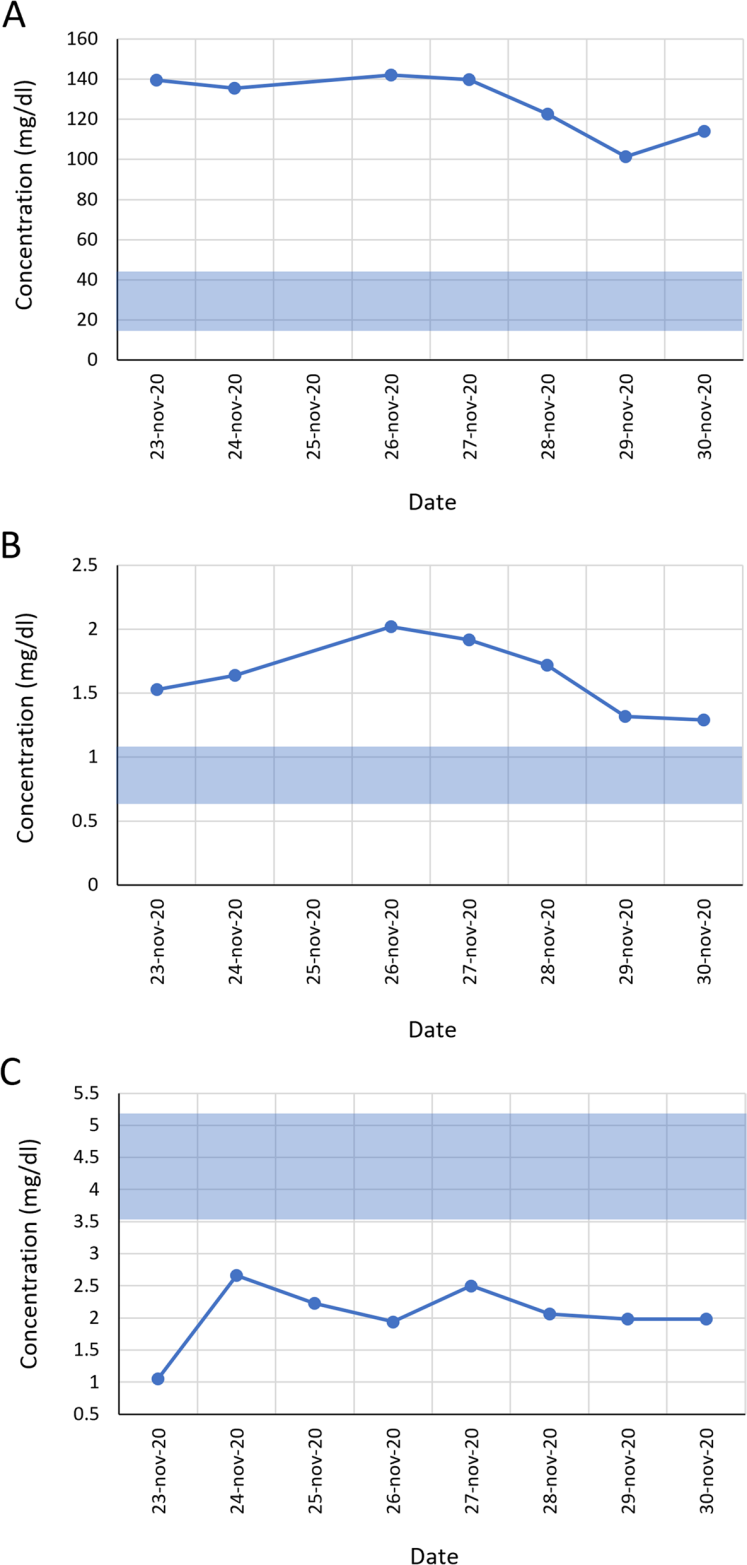
Renal profile tests. The graphs of (**A**) urea, (**B**) creatinine and (**C**) albumin values ​​measured during the patient’s hospitalization are shown. The solid blue bar represents the reference values ​​for each analyte

At least three indicators of renal dysfunction were also observed (Fig. 2): uremia (Fig. 2 A, mean urea values = 127.8 mg/dl), elevated creatinine levels (Fig. 2B, mean values = 1.6 mg/dl) and hypoalbuminemia (Fig. 2 C, mean albumin values = 2.1 mg/dl).

One day after admission to the emergency unit, an upper endoscopy was performed, and one of the findings was an esophageal candidiasis KPDSI III, for which fluconazole was administered (Table 2).

During the patient’s hospitalization, the levels of the inflammatory markers procalcitonin and C-reactive protein were measured. Regarding procalcitonin values, the following was obtained: on the day of hospital admission, it showed a moderate elevation (4.53 ng/ml); three days later, on November 26, the value increased significantly (26.69 ng/ml) even though the patient started with preventive treatment with ceftriaxone the day before. That same day a blood culture was performed and even without the laboratory result, it was decided to change the antibiotic to imipenem. The result of the blood culture (three days after the blood culture request) indicated growth of *Pseudomonas aeruginosa*, and treatment with imipenem was continued (indicated for the treatment of infection with *P. aeruginosa*). On the fifth day of hospitalization, the procalcitonin value continued to increase despite antimicrobial treatment (188 ng/ml).

Regarding the C-reactive protein (CRP) results, the value obtained on the day of hospital admission was within the normal parameter (8.44 mg/L). The next day, the concentration increased to 13.2 mg/L, which was not unexpected considering the underlying pathology (ICUC). On the fifth day of hospitalization, the concentration decreased to a value within the normal parameter (6.72 mg/L). During this period, the patient was administered with both non-steroidal and steroidal anti-inflammatory drugs.

In addition, other diagnostic support studies were performed during hospitalization due to the poor prognostic. In accordance with the clinical evolution, computed tomography scans were performed in different anatomical areas, the findings of which are described in Table 2. These studies, together with the laboratory results, revealed a refractory shock caused by a progressive organ deterioration (renal and neurological), requiring a double-vasopressor support, oxygenation, and ventilation.

**Table 2:** Clinical examinations

	Examination		
	Endoscopy	Chest, abdomen, and pelvis CT^a^	Skull CT^a^
*Date*	24-nov	27-nov	30-nov
*Findings*	• Esophageal candidiasis KPDSI III^b^ • Hyperemic pangastropathy • Erythematous bulboduodenitis • No evidence of active or recent bleeding	• Data on a pneumonic inflammatory process with multiple foci, probably due to atypical germs • Bilateral pleural effusion with posterobasal atelectasis • Simple liver cyst in segment VIII • Left renal calcification at the upper renal border • Right intramural myomatosis • Incipient data of degenerative disease of the thoracic spine • Soft tissue edema	• No evidence of acute intra- and / or extra axial lesions by this imaging method • Bilateral fronto-temporal chronic subdural hematomas (hygromas) • Cortico-subcortical atrophy • Sellar arachnoidocele grade II^c^

^a ^*CT * Computed tomography

^b ^Kodsi Classification of candida esophagitis

^c ^Empty sella syndrome classification

Computed tomography scans suggested a possible SARS-CoV-2 infection; therefore, on November 28 (day 5 of hospitalization), a COVID-19 test as well as D-dimer and ferritin determination were conducted. Nasopharyngeal and oropharyngeal swabs were performed, and the sample was tested for SARS-CoV-2 by using GeneFinder™ COVID-19 Plus Real*Amp* Kit, showing a positive result for COVID-19, with a high viral load evidenced by the quantification cycle (Cq) values: 16.4 for viral gene E; 17.17 for the viral gene N; 17.32 for the viral gene RdRp; and 27.7 for RNAse P housekeeping gene. D-dimer and ferritin levels were both higher than the reference values (1316 µg/l and 518.9 ng/ml respectively).

After considerable systemic deterioration, the patient displayed cardiorespiratory arrest, without response, and died on December 1, after 8 days of hospitalization.

Due to the unusual manifestations that the patient showed during the course of the disease, and given the very fast and fatal outcome, we decided to analyze the sequence of the viral S gene in search of possible mutations. A PCR product of 4,119 bp containing the complete S gene was retrotranscribed and amplified with the SuperScript IV One-Step RT-PCR System (Thermo Fisher Scientific) using specific primers designed for that purpose: 5´-GGGGTACTGCTGTTATGTCTTT-3´, and 5´- CGCCAACAATAAGCCATCCG-3´. The PCR product was analyzed by agarose gel electrophoresis and sequenced using the fluorescent cycle-sequencing method (BigDye Terminator Ready Reaction Kit; Applied Biosystems) by automated Sanger technology with an ABI 3500 sequencer (ABI 3500 Series Genetic Analyzer; Applied Biosystems).

**Fig. 3:**
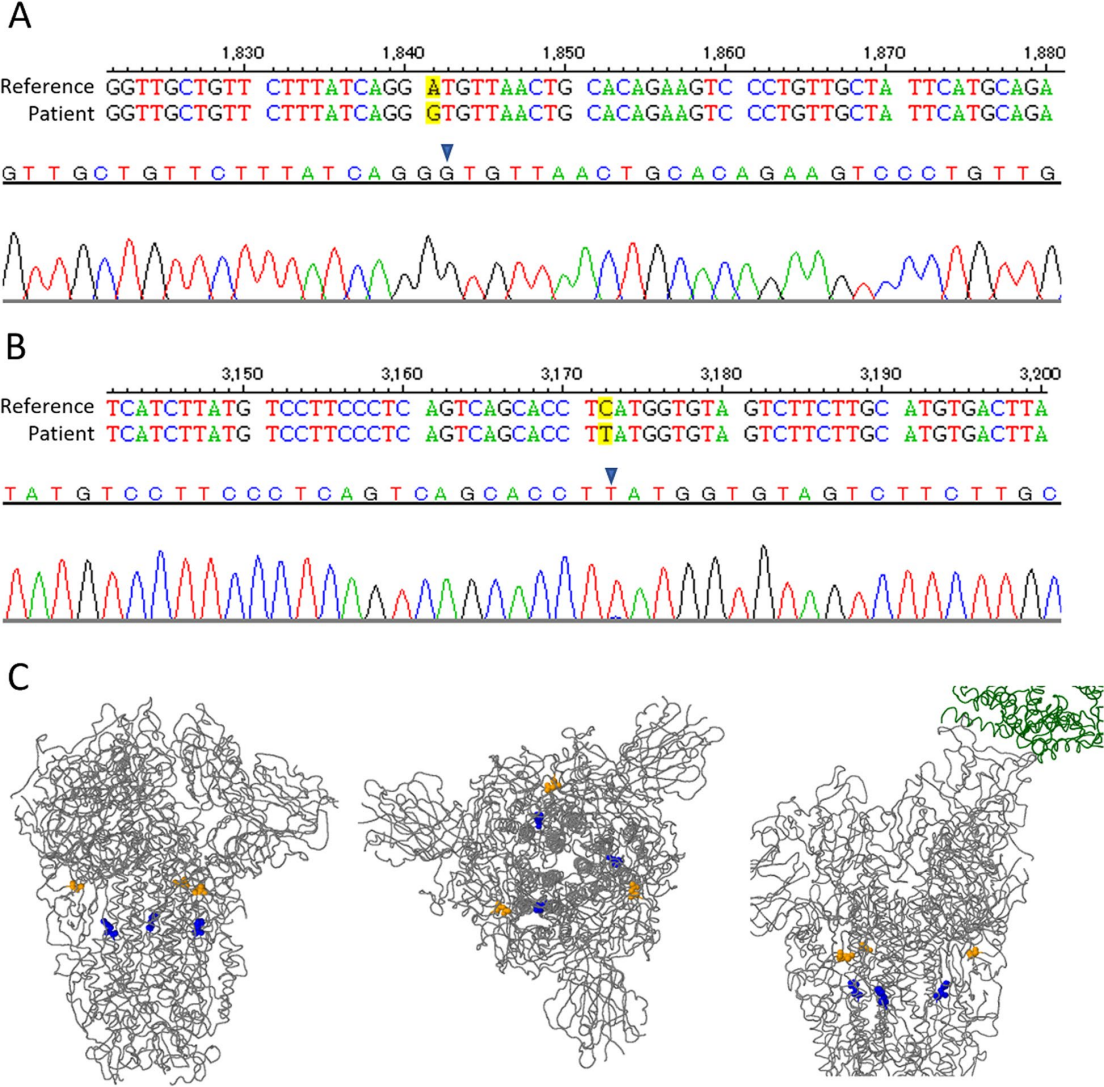
Sequencing and identification of mutations in the viral S gene. Comparisons of the reference sequence of the S gene and the sequence obtained from the patient’s oropharyngeal and nasopharyngeal swab are shown, at the sites where the two mutations were found. **A** The change of an adenine for a guanine at position 1841 (A1841G) is highlighted in yellow, and the electropherogram obtained where this mutation was found (black arrow) is shown. **B** The change of a cytosine for a thymine in position 3172 (C3172T) and the electropherogram where this mutation was found. **C** Three-dimensional structural visualization of the viral S protein with the amino acid changes identified, shown as colored balls: mutation D614G in yellow and mutation H1058Y in blue. Lateral view (left), upper (center) and lateral view bound to the ACE-2 receptor (in green, right)

Sequence analysis was performed using the DNASIS MAX software (Hitachi), finding two nucleotide differences compared to the reference SARS-CoV-2 genome sequence: the A1841G mutation (Fig. 3 A) causing a change of an aspartic acid to a glycine in the aminoacidic sequence (D614G); and the C3172T mutation (Fig. 3B) causing a change of a histidine to tyrosine in the aminoacidic sequence (H1058Y). The localization of the mutations in the S protein were analyzed in comparison to the reference sequence, and tridimensional graphics were generated with the CoVsurver tool for mutation analysis of hCoV-19 at the Global Initiative for Sharing All Influenza Data (GISAID) database (https://www.gisaid.org/epiflu-applications/covsurver-mutations-app/), finding both mutations in regions not related to the receptor binding domain (Fig. 3 C).

## Discussion and conclusions

In our institution, throughout the pandemic many cases of COVID-19 were treated, however, the rest of the patients (with other diseases) were not neglected. For this purpose, two different emergency rooms were installed: one for respiratory emergencies (for patients with suspected COVID-19), and another for non-respiratory emergencies. The patient referred to in this report attended the latter, as she had no respiratory symptoms and no suspicion of SARS-CoV-2 infection.

Considering the pathological history of the patient and her gastrointestinal symptoms, a case of inflammatory bowel disease was considered. Immediate treatment strategy was focused on the gastrointestinal symptoms, with a fluid and electrolyte replacement therapy to stabilize the patient and be able to transfer her to the specialty area. Once in the gastroenterology unit, specific treatment was started for the pathology presented, including fluconazole after being diagnosed with esophageal candidiasis (a very common infection in omeprazole-treated patients) by an upper endoscopy one day before the hospitalization. However, and despite the efforts, the patient did not evolve favorably.

Due to the lack of response to treatment, several laboratory and clinical studies were carried out for differential diagnoses. On November 26 (day 3 of hospitalization) a blood culture was performed, as the elevated procalcitonin concentration in serum suggested a bacterial infection. At the same time, treatment with imipenem was started. On November 27, for the first time the patient showed a respiratory symptom: dyspnea. The CT scan examination revealed a pneumonic inflammatory process, leading to the first suspicions of a SARS-CoV-2 infection, even though the patient did not show the classic symptoms of the disease. On November 28, D-dimer and ferritin levels were analyzed, both displaying elevated concentrations, and the suspicions were confirmed by a RT-qPCR test on November 29, finding high SARS-CoV-2 viral loads. By that time, the patient was showing intercostal retractions and spontaneous seizures.

The electrolyte disorder and the metabolic acidosis identified at hospital admission can be explained by the common gastrointestinal symptoms associated with ICUC, although it is also known that gastrointestinal disorders such as anorexia and diarrhea can occur in patients with COVID-19 [6, 7]; therefore, it is difficult to elucidate whether the symptoms were due to the patient’s chronic disease or as a result of SARS-CoV-2 infection. Concerning electrolyte imbalance, after the replacement therapy the concentration of some electrolytes returned to the reference range, however, phosphorus, chloride and calcium values always remained outside the normal range. This was undoubtedly due to the existing kidney damage at the time of admission (Table 1) [8, 9].

Regarding the metabolic acidemia, it is important to point out that, although it was compensated by adjusting the CO_2_ levels, the serum bicarbonate concentration was always below the reference value. This data, together with the elevated urea and creatinine values ​​(Fig. 2 A and 2B) also prove the acute kidney damage suffered by the patient. Kidney damage has been described within the complications developed by COVID-19 patients, in fact, the evident kidney damage in the patient is associated with severe COVID-19, and decreased survival rates [10–14].

The patient’s hypoalbuminemia throughout the whole hospitalization time (Fig. 2 C), and the elevated D-dimer and ferritin concentrations are additional risk factors for a severe disease course and a poor prognosis [15–17]. Figure 1 highlights the parameters of percentage and total count of altered blood cells measured during the hospital stay. Regarding neutrophils and monocytes, it is known that there is an association between these elevated values ​​and the severity of the disease [15, 16].

An acute lymphocytopenia has been seen in the blood of many. individuals with acute SARS-CoV-2 and is correlated with severe.

clinical outcome. This was the case of our patient, showing low lymphocyte count and percentage throughout the hospitalization time (Fig. 1B). The mechanisms that underlie lymphocytopenia are unclear but could reflect impaired lymphocyte proliferation, apoptosis, or extravasation into tissue [18].

Even though the patient showed all the clinical symptoms related to the pathological history of ICUC, the death was surely not due to this pathology, since the incidence of mortality reported is very low [19, 20]. Although it was not the main cause of death, the inflammatory chronic state could have increased the severity of the organ failure after SARS-CoV-2 infection, even in the absence of respiratory symptoms. In the same sense, SARS-CoV-2 infection could have participated in the poor response to treatment of the underlying disease and subsequent infections.

As for the mutations found in the viral S gene, the first one (D614G) is a mutation that emerged very early in the pandemic and quickly displaced the original sequence due to the biological advantages that this change confers, such as higher infectivity [21]. Today, this change is found in more than 99% of all the sequences reported throughout the entire pandemic. It is therefore not surprising that such a change was found. However, the second mutation (H1058Y) does draw attention as it is a rare mutation found in only around 0.02% of reported sequences worldwide (https://www.gisaid.org/; [22–24]. Interestingly, this change is not located in the receptor binding domain of the S protein, responsible of the recognition of the ACE-2 receptor (Fig. 3 C). It is necessary to further analyze whether this mutation gives a biological advantage to the virus or whether it generates a difference in any of the biological processes of the infection, such as the affinity for the host receptor (ACE-2 or other molecules), the infective capacity, replication capacity, immune system evasion, etc. In addition, it should be noted that, for this particular case, only the S gene sequence was analyzed, so we cannot rule out the existence of other mutations in any of the other viral genes.

It is also interesting to note that the patient did not show respiratory symptoms, even though she did have an active infection in the respiratory tract, confirmed by the RT-qPCR test from oropharyngeal and nasopharyngeal exudates, with a high viral load in these tissues. It is likely that the clinical history of the patient, as well as her own genetic characteristics, could have promoted a different response to the SARS-CoV-2 infection, including the rapid organ failure and fatal outcome, despite the absence of comorbidities that are frequently associated with a poor prognosis, such as diabetes, obesity, or hypertension. That is why atypical cases of COVID-19 should not be underestimated, nor should patients with no comorbidities or symptoms associated with the severity of the disease.

## Data Availability

The datasets used and/or analyzed during the current study are available from the corresponding author on reasonable request.

## Abbreviations

COVID-19: Coronavirus disease 2019
SARS-CoV-2: Severe Acute Respiratory Syndrome Coronavirus 2
ACE-2: Angiotensin Converting Enzyme 2
ICUC: Idiopathic Chronic Ulcerative Colitis
AKIN: Acute Kidney Injury Network
CONUT: Controlling Nutritional status
BMI: Body Mass Index
CT: Computed tomography
Cq: Quantification cycle
